# The effects of COVID-19 fear and anxiety on symptom severity, sleep quality, and mood in patients with fibromyalgia: a pilot study

**DOI:** 10.1186/s42358-021-00200-9

**Published:** 2021-06-30

**Authors:** Damla Cankurtaran, Nihal Tezel, Buse Ercan, Sadik Yigit Yildiz, Ece Unlu Akyuz

**Affiliations:** Department of Physical Medicine and Rehabilitation, University of Health Sciences Diskapi Yildirim Beyazit Training and Research Hospital, Altındag, 06080 Ankara, Turkey

**Keywords:** Fibromyalgia, COVID-19, Fear of COVID-19 scale, Coronavirus anxiety scale, Symptom severity

## Abstract

**Background:**

During the COVID-19 pandemic, individuals faced psychological stress caused by fear and anxiety due to the high transmission and mortality rate of the disease, the social isolation, economic problems, and difficulties in reaching health services. Fibromyalgia (FM) is a chronic centralized pain sensitivity disorder. Psychological, physical and/or autoimmune stressors were found to increase FM symptoms. This pilot study aimed to evaluate the COVID-19 fear and anxiety level, and to examine their effect on disease severity, sleep quality, and mood in FM patients compared to control group.

**Methods:**

This pilot study conducted as a cross-sectional study, and included 62 participants. Participants were divided into two groups: FM patient group (*n* = 31) and control group (*n =* 31). Symptom severity, sleep quality, and mood were determined using the Revised Fibromyalgia Impact Questionnaire (FIQR), Pitsburg Sleep Quality Index (PSQI), and Hospital Anxiety Depression Scale (HADS), respectively. In order to evaluate the level of COVID-19 fear and anxiety, the Fear of COVID-19 Scale (FCV-19S) and Coronavirus Anxiety Scale (CAS) were used compared to control group.

**Results:**

FIQR, PSQI, HAD-A, HAD-D, FCV-19S and CAS scores were significantly higher in the FM group (*p* = 0.01). A positive significant correlation was found between FCV-19S and CAS results and FIQR, PSQI, and HAD-anx results in FM patients (*p* < 0.05).

**Conclusion:**

This pilot study showed that, the individuals with FM can be more affected by psychological stress, and this situation negatively affects the symptom severity, sleep quality, and mood in FM patients, so these patients should be closely monitored in terms of psychological stressors and their effects during pandemics. More studies with more participants are necessary to describe the challenges lived by fibromyalgia population.

## Introduction

The World Health Organization (WHO) announced the start of an epidemic related to the novel coronavirus disease (COVID-19) that caused viral pneumonia in Wuhan, China, in December 2019. Since then, COVID-19 has spread all over the world and WHO declared it to be a pandemic in March 2020 [1]. Previous studies have shown that individuals’ well-being was adversely affected by prior pandemic periods [2–4]. During the COVID-19 pandemic, individuals faced psychological stress caused by fear and anxiety due to the high transmission and mortality rate of the disease, the social isolation which resulted from quarantine to prevent rapid transmission, economic problems, and difficulties in reaching health services [5, 6]. Previous studies have found an increase in mental disorders such as anxiety, depression, and post-traumatic stress disorder during the COVID-19 pandemic [5–7].

Fibromyalgia (FM) is a chronic centralized pain sensitivity disorder with widespread, wandering, increasing-decreasing pain attacks accompanied by fatigue, sleep disturbance, mood disorders, and somatic symptoms [8]. In controlled studies in the literature, psychological, physical and/or autoimmune stressors were found to increase FM symptoms [9]. Anxiety and psychological stress cause an increase in symptom severity in FM patients and adversely affect their quality of life [10, 11].

There are studies in the literature examining the effect of the lockdown period on FM symptoms but there is no study examining the effect of COVID-19 fear and anxiety in FM patients (disease severity, sleep quality, and mood) [9, 12]. Therefore, our study aimed to evaluate the COVİD-19 fear and anxiety level, and to examine their effect on disease severity, sleep quality, and mood in FM patients.

## Materials-method

### Ethical statement

This study was approved by the local institutional ethics committee of our hospital (Approval number: 102/17) and was conducted in accordance with the Declaration of Helsinki guidelines. Signed informed consent was obtained from each participant prior to data collection.

### Study design and participants

This pilot study conducted as a cross-sectional study, and included 62 participants. Participants were divided into two groups as the FM patient group (*n* = 31) and control group (*n =* 31). The FM group included patients between the ages of 18–65, who applied to the physical medicine and rehabilitation outpatient clinic between December 15, 2020, and February 1, 2021, who had been diagnosed with primary FM for more than 1 year according to the 2016 American College of Rheumatology criteria, and who had not been changed drug or dose for 3 months, and who had been clinically stable for at least 3 months. Patients with secondary FM or was changed drug or dose within 3 months, mood disorders, history of medication with any antidepressant or anxiolytic drugs, advanced musculoskeletal problems, additional rheumatologic diseases, COVİD-19 infection history, severe metabolic, endocrine, cardiovascular, pulmonary, genitourinary, gastrointestinal, progressive or non-progressive central or peripheral neurological diseases; and patients who underwent some type of therapy like yoga, meditation or other alternative therapies were not included in the study. The control group was selected from healthy volunteers (relatives or clinic staff) with no manifestation of disease and ages between the ages of 18–65.

### Data collection

Data related to demographic characteristics such as age, gender, height, weight, body mass index, professions, educational status, and marital status were obtained from the participants. In addition, symptom severity, sleep quality, and mood were determined using the Revised Fibromyalgia Impact Questionnaire (FIQR), Pitsburg Sleep Quality Index (PSQI), and Hospital Anxiety Depression Scale (HADS), respectively. In order to evaluate the level of COVID-19 fear and anxiety, the Fear of COVID-19 Scale (FCV-19S) and Coronavirus Anxiety Scale (CAS) were used.

### Revised fibromyalgia impact questionnaire (FIQR)

FIQR was used to evaluate FM physical functionality and the severity of its symptoms. The questionnaire focuses on three areas including function, overall impact, and symptoms. The total score ranges from 0 to 100, and higher scores indicate worse functionality and increased symptom severity [13].

### Hospital anxiety depression scale (HADS)

HADS is a scale developed specifically to evaluate mood disorders such as anxiety and depression. The scale includes a total of 14 questions, 7 of which belong to the anxiety subscale, and the remaining 7 questions belong to the depression subscale. Each question is scored between 0 and 3, and both the depression and anxiety subscale scores range from 0 to 21. Higher scores indicate more severe anxiety and depression [14].

### Pitsburg sleep quality index (PSQI)

The PSQI is a scale consisting of 19 self-reported questions and 7 items that evaluate sleep quality. The total score of the survey is obtained from the sum of the 7-item scores. The scores range from 0 to 21, and lower scores indicate better sleep quality [15].

## Fear of COVID-19 scale (FCV-19S)

FCV-19S is a short, 7-question questionnaire developed to evaluate the level of fear and anxiety related to COVID-19 virus*.* Each question is evaluated with a five-point Likert type scale (1 = strongly disagree to 5 = strongly agree) [16]. The Turkish adaptation of the questionnaire was made by Satıcı et al. [17]. The cut-off value in FCV-19S is determined as 16.5, and scores higher than this value have a significant predictive power on anxiety, health-related anxiety, and post-traumatic stress disorder [18].

### Coronavirus anxiety scale (CAS)

CAS is another 5-question questionnaire developed by Lee et al. to evaluate the psychological effects of the pandemic period. Validity and reliability checks of the Turkish adaptation of the scale have been conducted Each question is scored using a 5-point time anchored scale (0 ¼ not at all to 4 ¼ nearly every day over the last 2 weeks) [19, 20].

### Comparisons

COVID-19 fear and anxiety were compared between the FM and control groups, and the relationship between the level of COVID-19 fear and anxiety and FM symptom severity, sleep quality, and mood was investigated in the FM group.

### Statstical analyses

`Data analyses were conducted using Statistical Package for the Social Sciences (SPSS 22.0 for Windows) software. The variables were investigated using visuals (histograms and probability plots) and Shapiro-Wilks test to determine whether they are normally distributed. In reporting descriptive statistics, data were expressed as mean ± standard deviation (SD) and median (minimum-maximum) for continuous variables, and as frequencies and percentages (%) for nominal and categorical variables. The χ^2^ tests, Fisher’s exact or Likelihood ratio tests were used to compare nominal variables and categorical variables between the groups. In addition, the independent samples T tests and Mann-Whitney U tests were used to compare the continuous values between the groups. The relationship between FCV-19S, CAS and FIQR, PSQI, and HADS in the FM group were examined by Spearman correlation analysis. A *p*-value less than 0.05 (*p* < 0.05) was accepted as statistically significant.

## Results

No significant differences were found between the two groups in terms of age, gender, BMI, marital status, education level, occupation, and comorbidities (*p* > 0.05). Table 1 shows comparison of general characteristics of the participants of the two groups.

**Table 1:** Comprasion of general characterristics of participants between two groups

	FM group ***n*** = 31	Control Group ***n*** = 31	P
**Age (years)**			
*Median ± SD*	45.9 *± 9.33*	41.91 *± 9.17*	0.06^a^
**Gender** *n(%)*			
Female/male	28 (90.3)/3 (9.7)	26 (83.9)/5 (16.1)	0.71^b^
**BMI (kg/cm**^**2**^**)**			
*Median ± SD*	28.59 *± 5.22*	26.49 *± 3.55*	0.18^a^
**Education Status** *n(%)*			
İlliterate	2 (6.5)	0	
Primary school	12 (38.7)	15 (48.4)	0.13^c^
Middle school	7 (22.6)	2 (6.5)	
High school	5 (16.1)	5 (16.1)	
Univesity	5 (16.1)	9 (29.0)	
**Mariatal status** *n(%)*			
Marrage	22 (71.0)	20 (64.5)	
Single	7 (22.6)	9 (29.0)	0.85^c^
Divorcee	2 (6.5)	2 (6.5)	
**Professions** *n(%)*			
Unemployed /housewife	20 (64.5)	18 (58.07)	
Worker	7 (22.6)	5 (16.1)	0.28^c^
Student	1 (3.2)	0	
Officier	2 (6.5)	8 (25.8)	
Other	1 (3.2)	0	
**Comorbidities** *n(%)*			
No	20 (64.5)	25 (80.6)	
HT	2 (6.5)	3 (9.7)	
DM	2 (6.5)	2 (6.5)	0.20^c^
HT + DM	2 (6.5)	0	
Hipothyroidism	2 (6.5)	0	
Other	3 (9.7)	1	

*FM* fibromiyalgia, *SD* standard deviation, *BMI* body mass index, *HT* hypertension, *DM* Diabetes mellitus, ^a^ independent sample t test, ^b^ Fisher exact test, ^c^ Likelihood ratio test, bold values show *p* < 0.05

FIQR, PSQI, HADS-A, HADS-D, FCV-19S and CAS scores were significantly higher in the FM group compared to the control group (*p* = 0.01) (Table 2).

**Table 2:** Comprassion of results of Fibromiyalgia Impact Questionnare, Pitsburg Sleep Quality Index, Hospital Anxiety and Depression Scale, Fear of COVİD-19 Scale, and Coronavirus Anxiety Scele between two groups

	FM group ***n*** = 31	Control Group ***n*** = 31	p
**FIQR**			
*median (min-max)*	69.13 (25.22–94.22)	18.33 (10.95–73.98)	**0.01**^**a**^
**PSQI**			
*median (min-max)*	10 (5–15)	4 (1–14)	**0.01**^**a**^
**HADS-A**			
*median (min-max)*	9 (4–20)	6 (1–17)	**0.01**^**a**^
**HADS-D**			
*median (min-max)*	10 (1–17)	5 (1–18)	**0.01**^**a**^
**FCV-19S**			
*median ± SD*	20.96 *± 8.86*	14.67 *± 7.13*	**0.01**^**b**^
**CAS**			
*median (min-max)*	5 (0–18)	0 (0–18)	**0.01**^**a**^

*FIQR* Fibromiyalgia impact questionnare-revised, *PSQI* Pitsburg sleep quality index, *HADS-A* Hospital anxiety and depression scale-anxiety subscale, *HADS-D* Hospital anxiety and depression scale-depression subscale, *FCV-19S* Fear of coronavirus-19 scale, *CAS* Coronavirus anxiety scale, ^a^ Mann-Withney U test, ^b^ independent sample t test, bold values show *p* < 0.05

A positive significant correlation was found between FCV-19S and CAS results and FIQR, PSQI, and HAD-anx results in FM patients (*p* < 0.05) (Table 3). No significant correlation was found between the HAD-dep and FCV-19S and CAS results (*r =* 0.316, *p* = 0.084; *r =* 0.339, *p* = 0.062, respectively).

**Table 3:** Realiation between Fibromiyalgia Impact Questionnare, Pitsburg Sleep Quality Index, Hospital Anxiety and Depression Scale, and Fear of COVİD-19 Scale, Coronavirus Anxiety Scele in Fibromiyalgia Group

***n*** = 31	FCV-19S		CAS	
	r	P	r	p
**FIQR**	0.724	**< 0.01**	0.558	**0.001**
**PSQI**	0.416	**0.020**	0.439	**0.014**
**HADS-anx**	0.712	**0.001**	0.423	**0.018**
**HADS-dep**	0.316	0.084	0.339	0.062

*FIQR* Fibromiyalgia impact questionnare-revised, *PSQI* Pitsburg sleep quality index, *HADS* Hospital anxiety and depression scale, *FCV-19S* Fear of coronavirus-19 scale, *CAS* Coronavirus anxiety scale, *r* correlation cofficients, *p* values show statistical significance, bold values show *p* < 0.05, *p* values calculated with Spearman correlation test

A high level of positive significant correlation was found between FCV-19S and CAS results for all patients (*r =* 0.778, *p* < 0.001).

## Discussion

It was not find similar study the relates the effect of COVID-19 anxiety and fear on symptom severity, sleep quality, and mood in FM patients. The results of ongoing research revealed that the COVID-19 fear and anxiety levels in FM patients were higher than healthy patients. In FM patients, COVID-19 fear and anxiety were found to be associated with symptom severity, sleep quality, and anxiety level.

Although the cause of FM is still not fully understood, central sensitization in the central nervous system, which is associated with the development of increased sensitivity to pain signals, is the most accepted mechanism [21]. Although the main symptom of FM is pain, it is a heterogeneous picture that seriously disrupts the quality of life, including physical and mental fatigue, impaired sleep quality, headache, irritable bowel syndrome, cognitive disorders, and psychological problems [10]. The most common psychological problems in FM patients are anxiety and depression. Anxiety disorders are found in between 13 and 64% and depression in between 20 and 80% of FM patients [10]. In one study, 80% of FM patients reported poor sleep quality [22].

The whole world faced an unexpected pandemic in 2020, leading to negative psychological effects in the general population such as depression, stress, sleep disturbance, anger, and anxiety about one’s own health and the health of their relatives, phobias, and avoidance behavior [7]. Specifically, infected people, individuals with a high risk of infection (including the elderly, people with compromised immune function, and those living or cared for in public places), individuals with pre-existing medical or psychiatric illnesses, and those who had substance use problems were found to have an increased risk for adverse psychosocial outcomes [23].

During the pandemic period, new scales have been developed to evaluate the fear and anxiety associated with COVID-19 infection, which has a high transmission and mortality rate [16, 24, 25]. Among those scales, FCV-19S and CAS were used in our study. This study aimed to evaluate the level of fear and anxiety more accurately by using two separate scales and a significant correlation was found between the two scales.

Although the pandemic period has caused fear and anxiety in all individuals, previous studies have shown that those with pre-existing mood disorders were affected more by the negative psychological factors caused by the epidemic [26]. In our study, COVID-19-related fear and anxiety levels were found to be significantly higher in FM patients than the control group.

This study examined the effect of COVID-19 fear and anxiety on FM symptoms, sleep quality, and anxiety-depression levels. The results showed that COVID-19 fear and anxiety negatively affected FM symptom severity, sleep quality, and anxiety levels in FM patients.

In a study conducted by Hruschak et al., in which 150 patients with FM, chronic spine, and post-surgical chronic pain were included, patients’ self-reported pain severity was found to be higher after 4–8 weeks of social isolation compared to the pre-pandemic period. Furthermore, psychological factors such as anxiety, depression, sleep disorders, and stress were found to be associated with worse pain. In addition, the presence of FM was found to be an independent risk factor for greater pain during social isolation [27].

In a study comparing the symptom severity of FM patients in the pre-pandemic period with the lockdown period due to the COVID-19 pandemic, a significant difference was found in the total FIQR scores and pain, depression, and anxiety subdomains during the lockdown period compared to the pre-pandemic period. Additionally, a significant increase was found in the Generalized Anxiety Disorder-7 score during the lockdown period [9]. In a letter to editor, 67% of 32 FM patients reported that their general health status (well-being) worsened during the pandemic period [12].

Anxiety and depression are two closely related conditions [28]. However, our study found no relationship between depression levels and anxiety due to COVID-19. COVID-19 causes acute stress in the daily life of individuals. The severity of depression has been shown to be related to the duration of exposure to the stressor [29]. Given that our study is a cross-sectional study, more studies with longer follow-ups are needed to evaluate this relationship.

This pilot study is limited due to its small sample size and cross-sectional design.

## Conclusion

Particular attention should be paid to patients with previous psychological disorders such as FM patients during pandemic periods. Because these individuals are more affected by psychological stress than other individuals, and this situation negatively affects the symptom severity, sleep quality, and mood in FM patients. The patients should be closely monitored in terms of psychological stressors and their effects especially using telemedicine applications, and necessary precautions should be taken immediately. We still continue to see the effects of the COVID-19 pandemic. Therefore, more studies with more participants are necessary to describe the challenges lived by fibromyalgia population.

## Data Availability

Not applicable.
